# Behavioral and Neurodynamic Effects of Word Learning on Phonotactic Repair

**DOI:** 10.3389/fpsyg.2021.590155

**Published:** 2021-03-10

**Authors:** David W. Gow, Adriana Schoenhaut, Enes Avcu, Seppo P. Ahlfors

**Affiliations:** ^1^Department of Neurology, Massachusetts General Hospital, Boston, MA, United States; ^2^Department of Psychology, Salem State University, Salem, MA, United States; ^3^Athinoula A. Martinos Center for Biomedical Imaging, Massachusetts General Hospital, Charlestown, MA, United States; ^4^Harvard-MIT Division of Health Sciences and Technology, Cambridge, MA, United States; ^5^Department of Radiology, Massachusetts General Hospital and Harvard Medical School, Boston, MA, United States

**Keywords:** emergence, phonotactic, phonology, effective connectivity, magnetoencephalography, speech perception, word learning, rule

## Abstract

Processes governing the creation, perception and production of spoken words are sensitive to the patterns of speech sounds in the language user’s lexicon. Generative linguistic theory suggests that listeners infer constraints on possible sound patterning from the lexicon and apply these constraints to all aspects of word use. In contrast, emergentist accounts suggest that these phonotactic constraints are a product of interactive associative mapping with items in the lexicon. To determine the degree to which phonotactic constraints are lexically mediated, we observed the effects of learning new words that violate English phonotactic constraints (e.g., *srigin*) on phonotactic perceptual repair processes in nonword consonant-consonant-vowel (CCV) stimuli (e.g., /sre/). Subjects who learned such words were less likely to “repair” illegal onset clusters (/sr/) and report them as legal ones (/∫r/). Effective connectivity analyses of MRI-constrained reconstructions of simultaneously collected magnetoencephalography (MEG) and EEG data showed that these behavioral shifts were accompanied by changes in the strength of influences of lexical areas on acoustic-phonetic areas. These results strengthen the interpretation of previous results suggesting that phonotactic constraints on perception are produced by top-down lexical influences on speech processing.

## Introduction

There is a close relationship between the lawful patterning of speech sounds and the cognitive processes that allow listeners to recognize and produce spoken language. All human languages impose constraints on how speech sounds can be combined to form syllables and words. These phonotactic constraints are systematic, language-specific and productive (Chomsky and Halle, 1968; Berent, 2013). They capture the intuition of English speakers that *srib* would not make a good English word, but *slib* would. This patterning has critical implications for language processing. Infants leverage the lawful patterning of speech sounds to segment the speech stream and recognize words (Jusczyk et al., 1993; Mattys and Jusczyk, 2001). Adults show systematic biases toward recognizing, producing, and remembering unlawful forms in ways that are consistent with the constraints of their native languages (see Fromkin, 1971; Cohen and Massaro, 1983; Gathercole et al., 1999; Warker and Dell, 2006). While there is general agreement that there is a rich relationship between phonotactic structure and language processing, there is less agreement about the nature of this relationship. The purpose of this paper is to clarify the contribution of interactive spoken word recognition dynamics to phonotactic phenomena.

### Phonotactic Repair and Contrasting Accounts of Phonotactic Mechanisms

Phonotactic systems are self-reinforcing. Systematic perceptual, articulatory, and cognitive biases favoring lawful forms ultimately produce diachronic changes in phonological systems as the language processors of one generation provide the language models for the next (Reali and Griffiths, 2009). For this reason, any comprehensive understanding of phonological systems rests in part on understanding online mechanisms that enforce phonological regularity. In this work, we focus on perceptual phonotactic repair processes. In a key demonstration of perceptual phonotactic repair, Cohen and Massaro (1983) showed that listeners have a systematic bias toward interpreting ambiguous phonemes in nonword contexts in a way that produces allowable English onset consonant clusters. Given an /l/-/r/ continuum, listeners showed a bias toward /l/ responses to produce legal sl- clusters (versus illegal */sr/), but a bias toward /r/ in other contexts to produce legal tr- clusters (versus illegal */tl/). Subsequent work replicated these phenomena (Pitt, 1998; Breen et al., 2013) and identified illusory vowel epenthesis as an additional perceptual mechanism for repairing unlawful consonant clusters (Dupoux et al., 1999; Kabak and Isardi, 2007).

Generative linguistic theory provides one account of how this repair is enacted. It argues that language learners discover a set of abstract rules or constraints on phonotactic structure through exposure to the words of their native language, perhaps guided by innate learnability constraints (Chomsky and Halle, 1968). In approaches such as optimality theory (Prince and Smolensky, 2004) or harmonic grammar (Smolensky and Legendre, 2006), learners apply this knowledge of words to rank or weight a set of constraints that mediate all aspects of language usage, including the creation and adoption of new word forms. Generative accounts are not intended as processing models, but they do suggest a broad implicit model in which all speech input is evaluated against a grammar and repaired by modifications identified by the grammar. They primarily focus on the ability of grammars to account for patterns of attested structure, as well as intuitions about the acceptability of novel phonotactic structures. Several models have explored this approach using learning algorithms to construct phonotactic grammars from analyses of the lexicons of diverse human languages, including English, Shona and Wargamay (Clements and Keyser, 1983; Coleman and Pierrehumbert, 1997; Hayes and Wilson, 2008; Futrell et al., 2017). This work provides a plausible account for the key observations, including the finding that structures favored by linguistic analyses tend to be more common than less favored structures. In this approach, abstract constraints actively govern the patterning of the lexicon. Once in place, these constraints provide a powerful computational device for explaining listeners’ ability to generalize systematic linguistic intuitions to tokens outside of the lexicon (Albright and Hayes, 2003).

The influential Trace connectionist model of spoken word recognition (McClelland and Elman, 1986) provides an alternative to the generative linguistic theory account, attributing phonotactic constraints to top-down lexical influences on speech perception. The implication of this framework is that sensitivity to phonotactic structure in any aspect of language use results from top-down lexical influences in the processes that support that use. Top-down influences appear to serve the primary purpose of increased speed and robustness of spoken word recognition (Magnuson et al., 2018), but in doing so they introduce perceptual biases that enforce phonotactic order. McClelland and Elman (1986) addressed phonotactic repair directly. In simulations, they found that the Trace model produces biases towards lawful phonotactic forms through top-down lexical “gang” effects, in which partially activated words that resemble or overlap with an illegal form provide top-down support for an attested legal form. For example, there are no English words that provide top-down support for the sr- in /sri/, but weakly activated words with the phonetically similar pattern shr- (e.g., *shred*, *shrine*, and *shrimp*) produce top-down support for shr-. This activation in turn weakens activation of sr- through lateral inhibition.

### Neural Mechanisms That Support Phonotactic Processes

Both generative rule- or constraint-driven and lexically mediated approaches provide plausible accounts of phonotactic repair. Since phonotactic constraints and the structure of the lexicon are so intimately correlated in both approaches, discriminating between these accounts is challenging. Neuroimaging techniques offer several possible paths for distinguishing between rule- and similarity-driven processes. For example, one approach is to identify brain regions that support phonotactic processing and determine whether they co-localize with regions implicated in either rule-based processing or lexical wordform representation. Another approach is to study patterns of effective connectivity between brain regions and determine whether regions associated with rule- or lexical processing influence acoustic-phonetic regions.

A convergence of evidence from behavioral data, blood oxygenation level dependent (BOLD) imaging, and neuropathology clearly implicates the posterior middle temporal gyrus (pMTG) in wordform representations that mediate the mapping between sound and meaning (see reviews by Hickok and Poeppel, 2007; Gow, 2012). Gow (2012) argues that a similar convergence of evidence including findings from histology and magnetoencephalography (MEG) points to the role of the supramarginal gyrus (SMG) and inferior parietal lobe in the lexically mediated mapping between sound and articulation. Studies of artificial grammar learning and application, a frequent experimental surrogate for the kind of rule-driven processing proposed in generative linguistic theory, routinely produce activation or transcranial magnetic stimulation suppression effects involving the left inferior frontal gyrus or LIFG (see reviews by Fitch and Friederici, 2012; Uddén and Bahlmann, 2012). However, interpretation of the functional role of the LIFG and its subcomponents Brodmann areas BA 44/45 (Brocas’s area) remains complicated by its participation in a wide variety of linguistic and non-linguistic processes with proposed functions spanning cognitive control and selection, working memory, temporal abstraction and the movement of linguistic units (Thompson-Schill et al., 2005; Grodzinsky and Santi, 2008).

Several key results suggest a role of the rule-implicated LIFG in phonotactic processing. Vaden et al. (2011a) found a correlation between phonotactic frequency (how frequently subunits of spoken language occur within spoken words) and BOLD activation of the LIFG. This work relied entirely on legal (attested) phonotactic patterns in nonword contexts. Subsequent work by Berent et al. (2014) contrasted the BOLD responses to pseudowords that ranged from acceptable (e.g., *blif*) to increasingly ill-formed (*bnif*, *bdif*, and *lbig*) based on sonority profile in a syllable counting behavioral paradigm. This study leveraged the finding that listeners typically “repair” such clusters perceptually by inserting an epenthetic schwa to break up an illegal consonant cluster. For example, listeners might report hearing *bdif* as *bedif* (Pitt, 1998). Berent et al. (2014) found a positive correlation between illformedness and activation in bilateral posterior BA45, and a negative correlation between illformedness and activation in bilateral anterior BA45 in contrast with a rest condition. A near-infrared spectroscopy study by Rossi et al. (2011) found a greater hemodynamic response in the left hemisphere for legal vs. illegal German phonotactic patterns in sensors over temporal and frontotemporal regions that include the LIFG. While these studies provide converging evidence for sensitivity to phonotactic structure in LIFG, it is not clear what role LIFG plays in these tasks. A follow-up study by Vaden et al. (2011b) found that the correlation between phonotactic frequency and LIFG activation is modulated by manipulations of intelligibility. This led the authors to conclude that the frequency effects they observed in LIFG reflected task-specific downstream effects of word recognition difficulty rather than the direct effects of phonotactic factors on lexical processing. Obrig et al. (2016) offer a related interpretation of these results, suggesting that LIFG activation reflects lexical selection. This idea is developed independently in neuronal returning hypothesis of Matchin (2018). Based on a systematic review of the neuroimaging literature, Matchin (2018) argues that the left pars triangularis is part of a language-specific working memory system that performs general memory retrieval/attention operations. If LIFG activation reflects the application of phonological rules, the results of Rossi et al. (2011), Vaden et al. (2011a) and Berent et al. (2014) support rule- or constraint-driven accounts of phonotactic effects. However, if LIFG activation in those studies is the result of task-specific demands on working memory, selection, or cognitive control, the case for rule-driven processing is considerably weaker.

Several results link activation of presumed lexical areas to phonotactic manipulations. Ghaleh et al. (2018) found systematic differences in auditory pseudoword word-likeness judgments in a cohort of 44 aphasic patients with unilateral left hemisphere lesions when compared to unimpaired control subjects. Lesion-symptom mapping analyses provided significant statistical trends relating these changes to damage to the left angular gyrus (AG) and pMTG, but not LIFG. Additional voxel-based morphometric analyses showed stronger evidence linking the AG to these deficits. Ghaleh et al. (2018) suggest that the AG’s role in this task is to compare the incoming speech stream to lexical representations believed to be stored in the pMTG (Indefrey and Levelt, 2004; Hickok and Poeppel, 2007; Gow, 2012). A related study by Obrig et al. (2016) found a relationship between lesions to the SMG, AG, and anterior portions of the superior temporal gyrus (STG) and middle temporal gyrus (MTG) to reduced electrophysiological sensitivity to phonotactic violations.

The hemodynamic findings described above are broadly consistent with the results of a series of MEG-EEG studies that relied on high spatiotemporal resolution effective connectivity techniques to explore the directed dynamic interactions between brain regions that support phonotactic behavioral effects (Gow and Nied, 2014; Gow and Olson, 2015). This work was built on earlier work by Gow et al. (2008), which examined lexical influences on speech categorization in the Ganong paradigm (Ganong, 1980). Gow et al. (2008) found that behavioral evidence for top-down lexical effects on speech perception coincided with increased influence of the SMG on the left posterior STG (pSTG). The SMG is a brain region hypothesized to be associated with neural lexicon, whereas the left pSTG is believed to play a primary role in acoustic-phonetic representation and processing (Mesgarani et al., 2014). Gow and Nied (2014) found that the same neurodynamic signature, i.e., increased influence of SMG on pSTG, was associated with behavioral evidence for phonotactic influences on the categorization in a behavioral task modeled on paradigm of Cohen and Massaro (1983) paradigm. The same study also found increased influence of the pMTG on the pSTG in trials that produced behavioral responses consistent with phonotactic bias. In a related study exploring the neurodynamic bases of phonotactic frequency effects, Gow and Olson (2015) found that high phonotactic frequency words (words made up of frequently occurring phoneme patterns) elicited stronger top-down influences from SMG on pSTG than low phonotactic frequency items. Importantly, none of these studies showed a significant role of LIFG influences on pSTG.

### The Present Research and Predictions

The interpretation of the results of Gow and Nied (2014), Gow and Olson (2015), and Ghaleh et al. (2018) rests on the strong claim that influences from the SMG and pMTG are lexical. While this interpretation is consistent with evidence that these regions play a role in lexical representation (Hickok and Poeppel, 2007; Gow, 2012), it is possible that abstract phonotactic principles are independently co-represented in these regions. The immediate challenge then is to isolate lexical processes from potential grammatical processes behaviorally and neurally. To this end, we used a word learning paradigm to determine whether a specific lexical manipulation can influence phonotactic processes and whether such a manipulation influences hypothesized neural markers of top-down lexical influences on speech processing.

We taught participants a small set of meaningful novel wordforms with initial consonant clusters not found in familiar English words. We subsequently examined the behavioral and neural effects of word learning on nonsense syllables that contained those consonant clusters.

We used words with the /sr-/ and /∫l-/ contexts shown to produce phonotactic repair in Gow and Nied (2014). English allows words with /∫r-/ or /sl-/ onsets (e.g., *shrimp* or *sled*), but not words with /sr-/ or /∫l-/ onsets (Kenstowicz, 1994, p.258; Hayes and Wilson, 2008). Linguists have long recognized the existence of low-frequency phonotactic exception forms present in the lexicon due to language death or to loans from other languages that fail to generalize (Hock, 1991; Kurylowicz, 1995; Bybee, 2001; Albright, 2008). This suggests that learning a small set of words with anomalous consonant clusters in an experimental context should not introduce fundamental changes in the phonotactic constraints that govern the language as a whole. This creates a set of behavioral and neural predictions. If rule-based accounts of phonotactic processes are correct, the introduction of exceptions should have no effect on rates of phonotactic repair for stimuli that share these unlawful onset clusters, but otherwise differ phonologically. Any behavioral evidence for an effect of word learning on phonotactic repair would have to be attributed to the introduction of novel non-rule mechanisms reflected by new neural dynamics not present in the results of Gow and Nied (2014) results. However, if lexical mediation is responsible for the phonotactic repair, we predict that the introduction of new words with novel onset clusters should reduce rates of phonotactic repair for stimuli that share those onsets. If SMG and pMTG influences on pSTG reflect lexical influences on speech perception as hypothesized by Gow and colleagues (Gow et al., 2008; Gow and Segawa, 2009; Gow and Nied, 2014; Gow and Olson, 2015, 2016), word learning should produce changes in the strength of these influences. Furthermore, if lexical mediation is sufficient to account for phonotactic repair, we predict that word learning should not introduce neural dynamics unrelated to lexical access or control processes not found in Gow and Nied (2014).

## Materials and Methods

### Participants

Sixteen right-handed native speakers of American English, ages 20–40 were recruited in this study. None had discernable visual, motoric, or auditory deficits that could potentially influence task performance. None of the subjects reported fluency in or significant exposure to languages that allow either /sr/ or /∫l/ word onsets. All subjects provided written informed consent using a protocol approved by the MGH Institutional Review Board. Of these, four participants were tested but excluded from analyses due to equipment malfunction and recording issues (*n* = 2) or lack of a significant behavioral effect (*n* = 2). Twelve subjects were included in the final analysis [mean age 25.8 years (*SD* = 5.1), nine females]. Subjects were randomly divided between two familiarization training groups (discussed below).

### Stimuli

For the word learning (familiarization) portion of the protocol, subjects learned a set of names for photographs of 21 visually distinctive objects (Gogo’s Crazy Bones™ character pieces, see Supplementary Table S1). All object names were bisyllabic and composed of low phonotactic frequency nonword syllables drawn from stimulus set of Vitevitch and Luce (1999). Three of the object names had onset consonant clusters that are disallowed in English. For subjects in the sr-familiarization group, these were words with */sr/ onsets (*sradex*, *sraspar*, and *srigin*). For subjects in the shl-familiarization group, the same objects were paired with words with */∫l/ onsets (*shladex*, *shlaspar*, and *shligin*). Visual inspection of spectrograms and careful review of auditory tokens by two phonetically trained native speakers of American English confirmed that familiarization tokens were produced without repair by vowel epenthesis or shifts in fricative place of articulation. There were also 18 distractor items of low-frequency phonotactic sequences (derived from Vitevitch and Luce, 1999) with simple consonant onsets (e.g., *nezgeg*, *futneek*, and *mishpook*). These additional items were designed to draw attention away from the phonotactic markedness of the three experimental items and to minimize opportunities to draw rule-like generalizations about phonotactic patterning based on the overall training set. Initial training materials paired each photograph with a recording of the pronunciation of the object name spoken by a female talker, and, with the word in written form to reinforce the identity of illegal clusters and reduce potential perceptual repair by subjects during the familiarization task.

Stimuli for the phoneme categorization task used in the MEG-EEG session consisted of nonword consonant-consonant-vowel (CCV) tokens. These tokens were created by inserting a token from a five-step [s] – [∫] continuum at the beginning of /_lV/and/_rV/ contexts. Auditory stimuli were recorded by a male speaker at a sampling rate of 44.1 kHz with 16-bit sound and manipulated using PRAAT (Boersma and Weenink, 2019). These recordings consisted of isolated nonsense syllables spoken in American English by a male native speaker. The five-step fricative continuum was developed by performing weighted spectral averaging of the isolated /s/ and /∫/ sounds and equating for duration at 80 ms. Recordings of the syllables /le/, /re/, /li/, /ri/, /lʌ/, and /r ʌ/ equated to a duration of 300 ms were cross-spliced onto the end of the fricatives at ascending zero-crossings. All auditory stimuli were normalized for mean amplitude.

### Procedure

Prior to data collection, subjects were required to demonstrate mastery of the familiarization stimuli using an online studying and quizzing system.^1^ Participants learned words by studying online pairings of recordings of new words paired with pictures of unfamiliar objects. To minimize the effects of phonotactic repair, all sound recordings were paired with orthographic representations of new words. Training trials consisted of a combination of discrimination trials in which subjects chose an image that matched a new word, or a word that matched an object from two options, and identification trials in which they were shown an image and asked to type its name. Feedback was given after each trial during training with both tasks. Subjects completed a minimum of 30 min of training per day for the 2 days directly before their neuroimaging session. Subjects had to achieve a score of 100% on an online identification quiz administered without trial-by-trial feedback at least 24 h before the imaging session to continue participating.

During the MEG-EEG session, the subjects performed a delayed two-alternative forced-choice phoneme categorization task that was administered without feedback. They were not told that the task related to the words they had learned. Subjects were told that the phoneme categorization task would be followed by a test of word learning, however, no word learning test was administered. To reduce electrophysiological artifacts, subjects were instructed to maintain fixation on the screen in front of them and only blink immediately after responding to a trial. The categorization task consisted of 270 trials, which were randomly organized into three blocks of 90 trials each. Subjects were given several minutes to rest between blocks. Written instructions were presented at the beginning of each block. At the beginning of each trial, an auditory CCV stimulus of 380-ms duration was played over headphones. After a 500 ms inter-stimulus interval (ISI), lateralized visual response probes “S” and “SH” appeared on the screen. The lateralization of the “S” and “SH” visual response probes varied randomly on a trial-by-trial basis. Subjects were given a keypad with two buttons to respond using their left hand. We used a left-handed response to make it easier to dissociate right hemisphere activity related with the motor response from language processing activity that is predominantly associated with left hemisphere activity in right-handed subjects. They were instructed to press the button on the same side (right or left) as the visual probe that best corresponded to the preceding auditory stimulus. The visual probe disappeared as soon as the subject responded. The next trial began 500 ms after the button press.

To understand how word learning interfered with phonotactic repair processes, trials were separated into Trained and Naïve conditions (Table 1). The Trained condition consisted of CCV stimuli with potential consonant clusters (depending on fricative categorization) found in the word learning training set. The Naïve condition consisted of CCV stimuli with consonant clusters that did not occur in the training set. Only those trials in which subjects made non-repaired phoneme classifications (i.e., S response for sr-shr continuum items or SH response for sl-shl continuum items) were selected for the effective connectivity analysis to more directly target dynamics attributed to phonotactic processing. We focused on non-repaired trials (min 135 trials for any participant) to test the hypothesis that newly learned words introduced new top-down lexical influences on acoustic-phonetic processing introducing a bias for non-repaired forms in the same way that Gow and Nied (2014) hypothesized top-down lexical influences from existing words create a bias towards repair in their study.

**Table 1:** Experimental words learned during training and the associated Trained and Naïve condition test continua used during subsequent fricative categorization testing.

Familiarization stimuli	Trained condition continuum [unrepaired response]	Naïve condition continuum [unrepaired response]
*sradex*, *sraspar*, and *srigin*	*/sr/-/∫r/ [“S”]	*/∫l/-/sl/ [“SH”]
*shladex*, *shlaspar*, and *shligin*	*/∫l/-/sl/ [“SH”]	*/sr/-/∫r/ [“S”]

The phonotactically unrepaired response choice is shown in brackets.

Specifically, the Trained condition trials were those in which subjects in the sr-familiarization group heard a stimulus beginning with a sound along the /sr/-/∫r/ continuum and responded that they heard an “s” sound, along with those in which subjects in the shl-familiarization group heard a stimulus beginning with a sound along the /∫l/-/sl/ continuum responded that they heard an “sh” sound. The Naïve condition trials were those in which subjects who were not trained on words with /sr/ onsets heard a stimulus beginning with a sound along with the /sr/-/∫r/ continuum and responded that they heard an “s” sound, along with those in which subjects who were not trained on words with “shl” onsets heard a stimulus beginning with a sound along the /∫l/-/sl/ continuum and responded that they heard an “sh” sound (27 trials in each step in each condition).

Analyses were further limited to steps 2–5 (108 trials for each participant) in the unlawful to lawful fricative phonetic continua (/sr/-/∫r/ and /∫l-/sl/) because the goal of the neural analyses was to understand the mechanisms that alter repair as a function of word learning, and step 1 did not contribute to the robust overall behavioral word learning effect.

### MEG and EEG Data Acquisition

Magnetoencephalography and EEG data were simultaneously collected using a whole head Neuromag Vectorview system (MEGIN, Helsinki, Finland) in a magnetically shielded room (Imedco, Hägendorf, Switzerland). The system includes 306 MEG channels (204 planar gradiometers and 102 magnetometers), and a 70 channel EEG cap with nose reference and two electro-oculogram (EOG) channels to identify blink and eye-movement artifacts. MEG and EEG data were band-pass filtered between 0.1 and 300 Hz and sampled at 1000 Hz. Before testing, a FastTrack 3D digitizer (Polhemus, Colchester, VT) was used to determine the positions of anatomical landmarks (preauricular points and nasion), all EEG electrodes, four head-position indicator (HPI) coils, and over 100 additional surface points on the scalp for co-registration with anatomical MRI data. Using the HPI coils, the position of the head with respect to the MEG sensor array was measured at the beginning of each block.

### Structural MRI

Anatomical T1-weighted MRI data were collected for each subject with a 1.5T Avanto 32 channel “TIM” system using an MPRAGE sequence. Freesurfer (Dale et al., 1999) was used to reconstruct the cortical surface for each subject, as well as to identify skull and scalp surfaces. A spherical morphing technique (Fischl et al., 1999) was used to co-register the cortical surfaces across individual subjects.

### Cortical Source Estimation and ROI Identification

To reconstruct spatiotemporal distributions of task-related cortical activation, MRI-constrained minimum-norm source estimates for combined MEG and EEG data were created as described in Gow and Nied (2014) using the MNE software (Gramfort et al., 2014). The analyses focused on the 100–500 ms time window after stimulus onset based on the window of electrophysiological sensitivity to phonotactic violations shown in previous studies (Rossi et al., 2013; Gow and Nied, 2014; Steinberg et al., 2016). Regions of interest (ROIs) were defined by an algorithm relying on the similarity and strength of minimum-norm estimate (MNE) activation time series at each source space vertex over the cortical surface for the 100–500 ms period after stimulus onset. Estimated cortical activity averaged over all trials from each subject were transformed to the common average cortical surface, and the across subject averaged activation map was used to identify a set of ROIs that satisfied the statistical and inferential requirements of Granger causality analysis. The locations of ROIs were labeled based on their location with respect to Freesurfer’s automatic parcellation utility. The ROIs thus obtained were transformed back onto individual subjects’ cortical surfaces, and optimal individual vertices (cortical source elements) from each subject were selected as input to Granger analyses.

The process of ROI identification consisted of three steps (Gow and Nied, 2014). First, potential centroids for ROIs were identified by selecting vertices with mean activation over the 95th percentile during the 100–500 ms time window after stimulus onset. In order to maintain a conservative approach to source reconstruction, vertices located within 5 mm of local maxima were excluded. Second, the similarity of contiguous vertices – quantified by the Euclidean distance between their normalized activation functions – was compared by iterating through each potential centroid. If the similarity in the activation function of a vertex was within 0.5 SDs of an ROI centroid, then the vertex was included in the ROI. Defining regions of similar activation time course structure allowed for representative vertices for each ROI to be identified on an individual subject basis, therefore controlling for differences in source localization between subjects. Third, redundant ROIs – those with activation functions within 0.9 SDs of an ROI with a stronger (non-normalized) signal – were eliminated. This step was necessary to satisfy the Granger analysis assumption that all predictive information carried by each signal is unique.

### Granger Causality Analysis Using Kalman Filter

We measured effective connectivity using a Kalman-filter-based Granger causality analysis technique (Milde et al., 2010). Our application of this approach is described at length in Gow and Caplan (2012). The Kalman filter approach addresses the noise in the MEG signals as well as Granger causation analysis’s assumption of signal stationarity. It also allows for the Granger causality measure to be tracked at each time point by estimating coefficients for time-varying multivariate autoregressive (MVAR) prediction models.

The Kalman filter-based Granger analysis was applied to the MNE activation time series data averaged over trials separately for each participant and condition for each ROIs. The time series from all ROIs were passed through the Kalman filter to generate the full multivariate autoregressive (MVAR) model predictive of the activity in a single ROI. For each ROI, a counter-models omitting one other (potentially causal) ROI at a time were created. The five samples preceding each time point were used to determine a basis for the following time point at each step of the Kalman filter. This model order of five was heuristically assigned because Akaike and Bayesian Information Criteria failed to determine a single optimal model order. The Kalman filter converged within about 100 ms, so the model was computed over time from 0 to 500 ms to cover the 100–500 ms time window of interest.

The Granger Causality Index (GCi) was computed at each point in time (Milde et al., 2010) for every potential directed interaction between ROIs in each condition. GCi is defined as the logarithm of the ratio of the standard prediction error in the model omitting an ROI containing a potentially causal signal vs. the full MVAR model. For each pair of ROIs, if the model omitting the potentially causal ROI has a greater standard prediction error than the model that includes it (the full MVAR model), then it could be assumed that the potentially causal ROI carries unique predictive information and therefore is said to Granger-cause changes in the other ROI.

A threshold value for the statistical significance of the GCis was determined using a bootstrapping method (Milde et al., 2010). For each condition and time point, 2000 trials of data were reconstructed from the matrices of the full MVAR model, eliminating one hypothesized causal ROI at a time and randomizing the residuals. For each directed ROI to ROI interaction for each point in time, an independent distribution of GCis was established to assign probability estimates to each computed GCi value. The strength of the Granger causality was assessed by counting the number of time points within the 100–500 ms post-stimulus time window that achieved the significance threshold of *p* < 0.05. To compare the Trained vs. Naïve conditions, a binomial test (Tavazoie et al., 1999) was performed on the difference in the number of time points that achieved the significance threshold (*p* < 0.05) in two conditions.

## Results

### Behavioral Results

**Figure 1:**
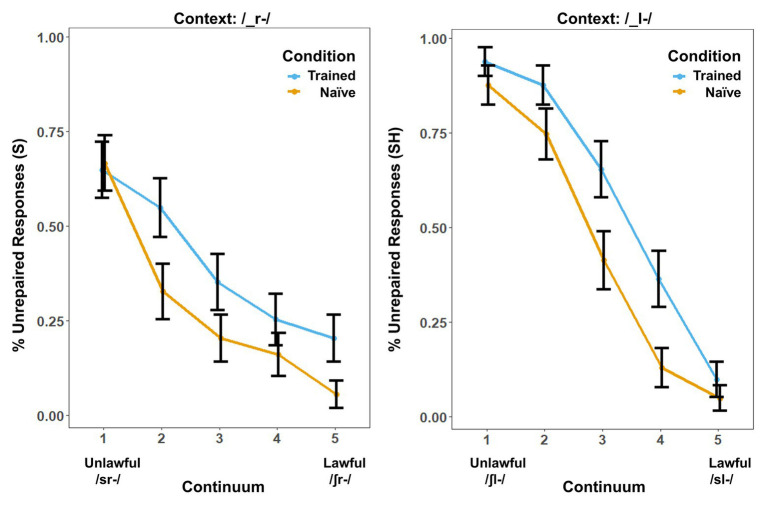
Behavioral results. Percentage of trials in which the subjects’ responses produced onset clusters that were phonotactically unrepaired (illegal) in the /-_r-/ and /_l-/s contexts. Error bars show the SE. Results are broken down by context because context produced a significant main effect in addition to the effect of training condition.

Behavioral results showed a marked influence of word learning on rates of phonotactic repair (Figure 1). While the same /s/-/∫/ continuum was used in both contexts, the order of the steps was reversed in the /_l-/ context so that both contexts could be collapsed into comparable lawful-to-unlawful continua for both analyses. We used the lme4 (Bates et al., 2012) package in R to perform a logistic mixed-effects analysis of the relationship between phonotactic repair and training condition. We entered Condition (two levels: Trained vs. Naïve), Context (two levels: /_l-/ vs. /_r-/) and Step (four levels: Step 2–Step 5 of the continua) as fixed effects into the model. In pilot studies, we found that Step 1 did not show a significant effect of learning on phonotactic repair in either context. We attribute this to a floor effect. Step 1 was therefore eliminated from all behavioral and neural analyses to provide a more direct window on the influence of word learning on repair. As random effects, we had intercepts for subjects, as well as by-subject random slopes, for the effect of Condition. Values of *p* were obtained by likelihood ratio tests of the full model with the effect in question against the (null) model without the effect in question. The results showed significant effects of Condition [*χ*^2^(1) = 4.92, *p* = 0.026], Context [*χ*^2^(1) = 5.76, *p* = 0.016], and Step [*χ*^2^(3) = 572.49, *p* < 0.001]. There was a significant three-way interaction, [*χ*^2^(10) = 71.68, *p* < 0.001] and a statistically significant two-way interaction between the effects of context and step [*χ*^2^(9) = 71.7, *p* < 0.001] and condition and step [*χ*^2^(7) = 71.5, *p* < 0.001] but not between the effects of condition and context [*χ*^2^(7) = 7.2, *p* = 0.413] on phonotactic repair. The main effects and interactions involving Context reflect an overall preference for “S” responses that interacted with the reordering of the fricative continuum in /_l-/ versus /_r-/ contexts.

### Neural Results

**Figure 2:**
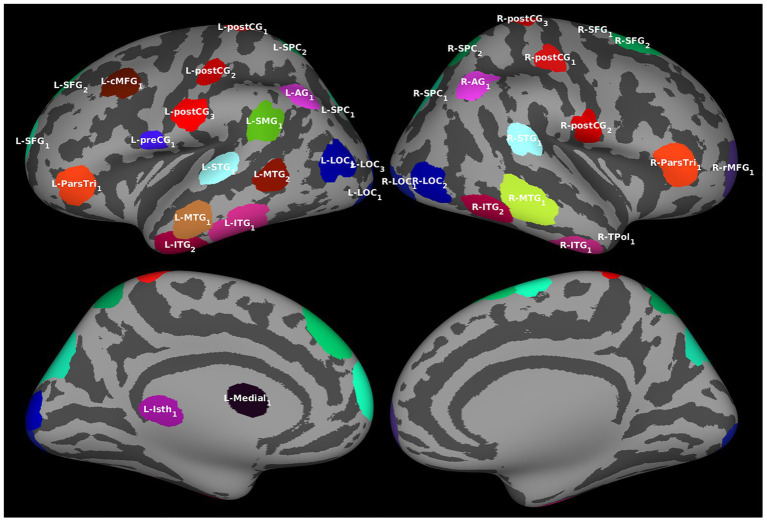
Regions of interest (ROIs). For the effective connectivity analyses, ROIs identified by algorithm based on the estimated cortical activation pattern. The ROIs are visualized over an averaged inflated cortical surface. Further details of the ROIs are given in Supplementary Table S2.

Regions of interest were determined by identifying clusters of vertices with similar temporal activation patterns in the source estimates averaged over all trials and filtering out those with redundant information. The procedure resulted in 39 ROIs (Figure 2; also see Supplementary Table S2), all of which were included in Granger analyses.

**Figure 3:**
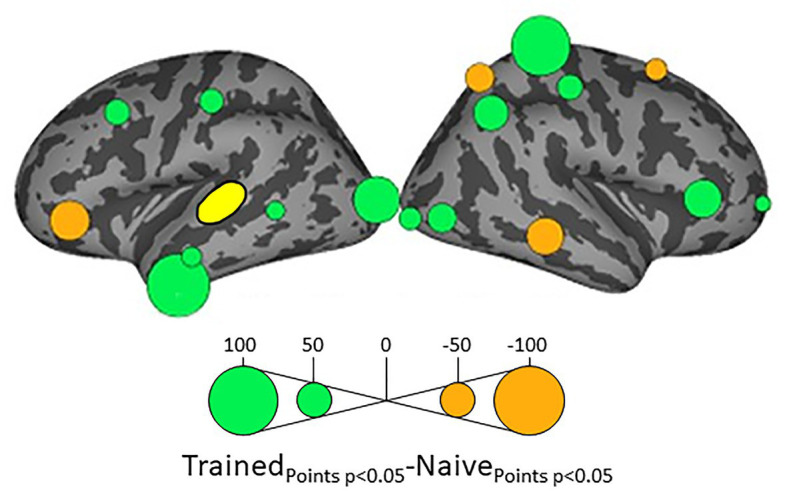
Differential influences on left posterior superior temporal gyrus pSTG (shown in yellow) by the other ROIs in the Trained and Naïve conditions. Green bubbles indicate significant differences (*p* < 0.05) in which influences were stronger in the Trained condition. Orange bubbles indicate significant differences in which influences were stronger in the Naïve (untrained) condition. Bubble radius indicates the difference in the number of timepoints during 100–500 ms post stimulus onset in which Granger Causality Index (GCi) reached the significance threshold of *α* = 0.05 in the two conditions. No significant results were found for the ROIs in the medial cortical surfaces.

**Figure 4:**
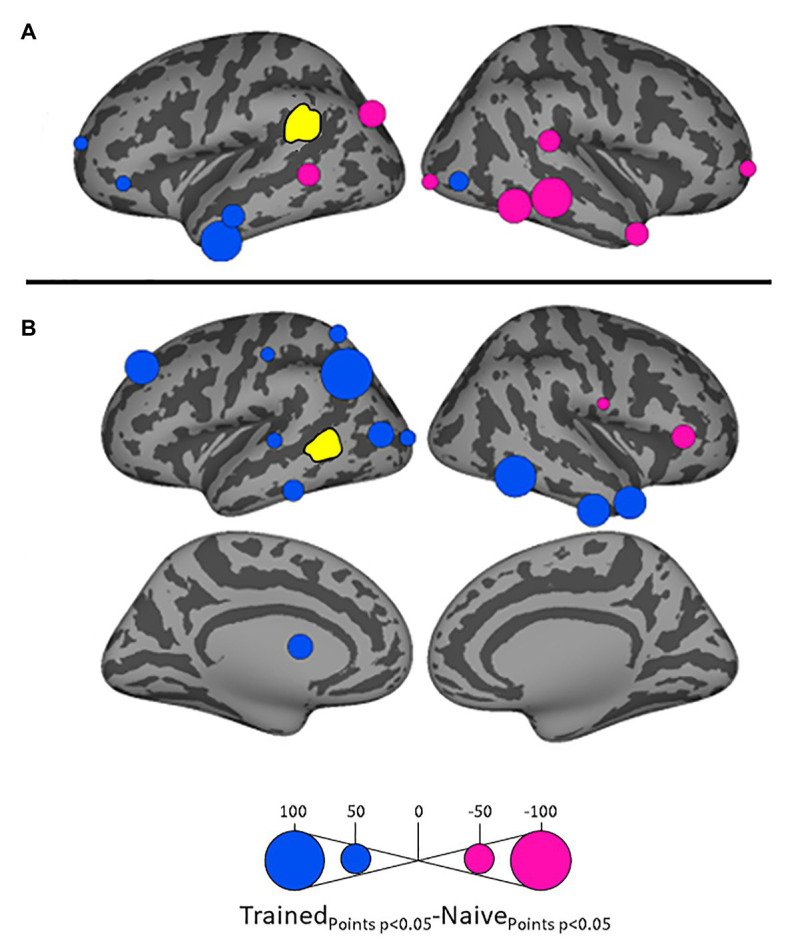
Differential influences by left supramarginal gyrus (SMG; **A**) and left posterior middle temporal gyrus (MTG; **B**, both shown in yellow) on other ROIs in the Trained vs. Naïve conditions. Blue and pink bubbles indicate significant differences (*p* < 0.05) in which influences are stronger in the Trained and in the Naïve conditions, respectively. No medial surfaces are shown in panel **(A)** because the left supramarginal exerted no significant influences on medial ROIs.

**Figure 5:**
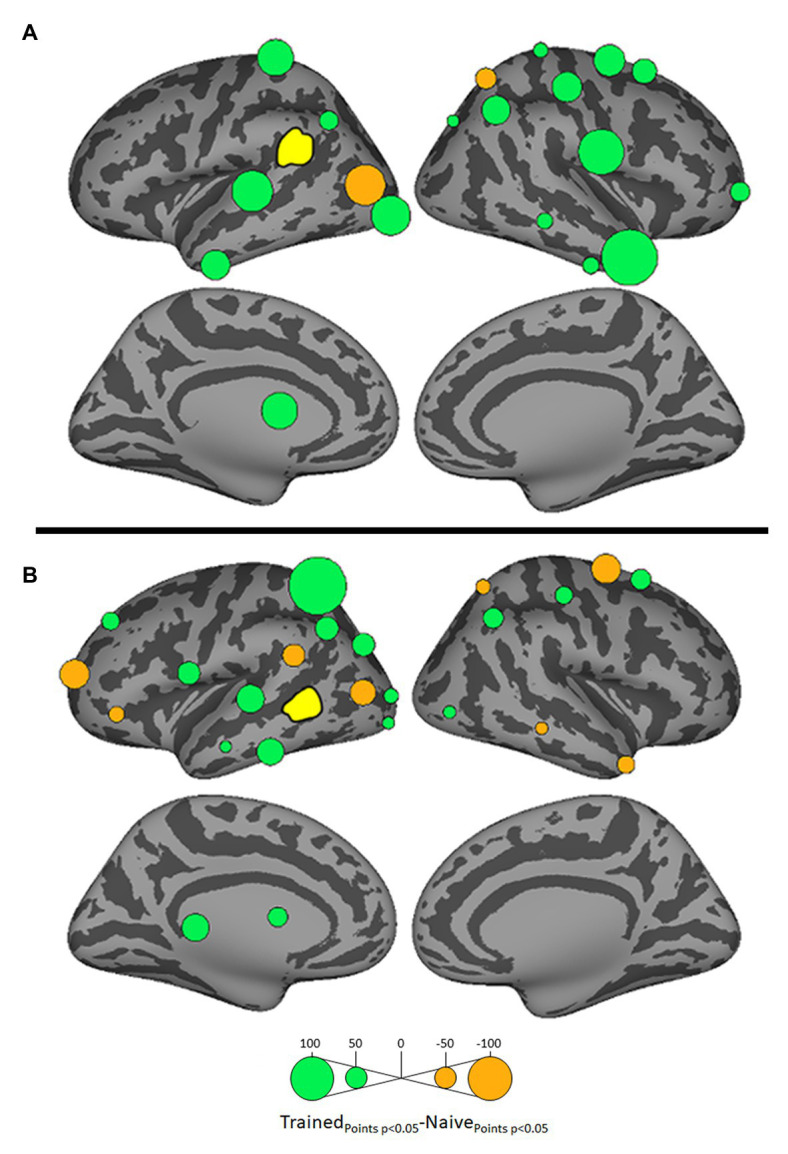
Differential influences on left SMG **(A)** and left posterior MTG (pMTG: **B**, both shown in yellow) by other ROIs in the Trained vs. Naïve conditions. Green and orange bubbles indicate significant differences (*p* < 0.05) in which influences are stronger in the Trained and in the Naïve conditions, respectively.

Because we hypothesize that phonotactic repair involves influences on acoustic-phonetic representation, our critical results center around influences on left pSTG (L_STG_1_), an area strongly associated with acoustic-phonetic representation (see Mesgarani et al., 2014), and top-down lexical effects on speech perception (Gow et al., 2008; Myers and Blumstein, 2008; Gow and Segawa, 2009; Gow and Olson, 2016). Figure 3 shows the relative influence of other ROIs on left pSTG activation between the Trained vs. Naïve conditions. In addition, because we were interested in the role of lexical influences on phonotactic processing we also examined the influence on and by two hypothesized lexical regions (Hickok and Poeppel, 2007; Gow, 2012) – the left SMG (L_SMG_1_) and pMTG (L_MTG_2_) – as a function of word learning. These analyses were done to examine potential indirect influences of wordform areas on pSTG *via* the network identified in Figure 3. These results are summarized in Figures 4, 5. All effects reported here were significant (α = 0.05) after correction for multiple comparisons using the false discovery rate (Benjamini and Hochberg, 1995).

Influences on left pSTG were significantly stronger in the Trained condition than in the Naïve condition for 13 of the other 38 ROIs (Figure 3). These included the left pMTG (L-MTG_2_; *p* < 0.001), which is implicated in the representation of wordforms (Hickok and Poeppel, 2007; Gow, 2012). It also included anterior portions of the left MTG (L-MTG_1_) and inferior temporal gyrus (L-ITG_2_) regions (both *p* < 0.001), which are associated with semantic processing of spoken words and familiar visual stimuli (Li et al., 1993; Patterson et al., 2007; MacGregor et al., 2012), and bilateral lateral occipital cortex areas (L-LOC_3_, R-LOC_1,2_; both *p* < 0.001). While the task did not explicitly call for semantic activation, we believe the influence of areas involved in semantic processing reflects the influence of representations associated with the small set of newly learned words and their visual associates on categorization performance. Notably, all three of these regions were themselves most strongly influenced by hypothesized wordform areas after word learning. The left pMTG and left inferior temporal gyrus were most strongly influenced by left SMG (both *p* < 0.001, Figure 4A), and the left lateral occipital cortex (LOC) was most strongly influenced by left pMTG (*p* < 0.001, Figure 4B) in comparisons between the Trained and Naïve condition. Such mediated influences of wordform areas on left pSTG as a function of word learning were widespread. Eight of the 13 regions that showed stronger influences on pSTG in the Trained condition were significantly influenced by one or both wordform areas. A ninth area, the more dorsal right postcentral gyrus (R-postCG_2_) showed stronger influences by left SMG (*p* = 0.034) that did not survive correction for multiple comparisons.

The two hypothesized wordform areas, left SMG and left pMTG, show increased importance in the Trained condition relative to the Naïve condition (Figure 5). Both areas showed increased feedforward influence from left pSTG after word learning (*p* < 0.001 for both). This is consistent with the formation of new form representations that align at least partially with the unlawful onsets of categorization stimuli. The wordform areas also show increased influence from the same network that drives the left pSTG afterword learning. Overall, 8 of the 13 regions that showed stronger influences on pSTG in the Trained condition were significantly driven by one or both wordform areas. In addition to the lexical areas, a number of regions involved in attention and control processes (Aron et al., 2003; Shomstein and Yantis, 2006; Vilberg and Rugg, 2008; Kim, 2010; Wild et al., 2012) including left caudal middle frontal gyrus, right rostral middle frontal gyrus, right pars triangularis, and right AG showed stronger influences on left pSTG in the Trained condition (*p* < 0.001 for all, Figure 3). The role of attention and control processes may reflect the need to devote additional effort to sustain input representations for newly established and perhaps less automatized processes related to learning new words or rules. The role of right pars triangularis deserves special comment. While the right pars triangularis shows increased activity during the application of novel syntactic rules (Musso et al., 2003), it not been implicated in studies specifically examining phonotactic phenomena (Rossi et al., 2011; Vaden et al., 2011a; Berent et al., 2014; Gow and Nied, 2014; Gow and Olson, 2015; Ghaleh et al., 2018). Independent evidence from negative priming and erroneous responses in naming tasks suggest that the right pars triangularis plays a general role in the inhibition of left hemisphere language networks associated with lexical access, especially when processing is challenging due to pathology or stimulus ambiguity (Aron et al., 2003, 2004; Snijders et al., 2009; Geva et al., 2012; Harvey et al., 2019).

Several postcentral gyrus regions also showed stronger influences on pSTG in the Trained condition (Figure 3). The largest effect involves the dorsal-most right postcentral gyrus region (R_postCG_3_). Its location aligns roughly with the region of the sensory homunculus associated with the left hand (Overduin and Servos, 2004). This suggests integration between acoustic-phonetic representation and sensorimotor activation associated with the left-hand button press response. More ventral bilateral middle postcentral gyri (postCG) regions also had a significantly stronger influence on left pSTG in the Trained condition (*p* < 0.001 for all). These areas are known to play a causal role in phonological processing, with special sensitivity to contrasts in place of articulation such as the /s/-/∫/ contrast (Schomers and Pulvermüller, 2016). Both of these areas received significantly stronger influences from left pMTG in the Trained condition (*p* < 0.0001, Figure 4B). We suspect that unrepaired trials in the Naïve condition reflect disengagement, and so this difference is due to a relative decrease in activity in the Naïve condition rather than a word learning-induced increase in activity in the Trained condition.

For four ROIs, the influence on pSTG was smaller in the Trained condition than in the Naïve condition: left pars triangularis (*p* < 0.001, Figure 3) and right MTG (*p* < 0.001), SFG (*p* < 0.002) and SPC (*p* < 0.001). Furthermore, three of these four regions, including left ParsTri, that showed stronger influences on pSTG in the Naive condition, were also significantly driven by one or both wordform areas. Given that analyses were limited to trials in which the subject’s response did not indicate phonotactic repair, these results cannot be interpreted as evidence that these regions play a differential role in processes that support repaired vs. non-repaired responses. Indeed, only one of the four, the right MTG, has been shown to differentially influence pSTG activation as a function of phonotactic phenomena in other studies (Gow and Nied, 2014; Gow and Olson, 2015). Evidence implicating the LIFG in lexical selection (Matchin, 2018), the right SFG in a response suppression (Hu et al., 2016), and the right SPC in control processes related to working memory (Koenigs et al., 2009) suggest that these reversals may indicate that subjects engaged in active suppression of representations in trials that produced phonotactic repair in the Naïve condition. Such effort may have been related to suppression of spuriously activated familiar foreign words such as the familiar loan word *schlep*, which contains illicit phonotactic patterns. Within an associative framework, exception loan words (e.g., *Sri Lanka* or *shlep*) fail to generalize robustly for several reasons. First, very few such words are familiar to English speakers, and those are often pronounced with repaired onsets (e.g., *Shri Lanka* or *slep*). Because exceptions are rare, they may not provide sufficient clusters of words with overlapping phonology to support robust gang effects. The strength of such gang effects would be further weakened by the fact that these loan words tend to have extremely low frequencies (Michigan Corpus of Spoken Academic English^2^), and thus may be less accessible than competing gangs with more frequent words with common onsets (e.g., *shrink* and *sled*).

## Discussion

The goal of this work was to determine whether interactions between language processing and phonotactic structure are mediated by processing interactions with the lexicon, and/or by the influence of abstract phonological rules governing possible or preferred phonotactic structures. Our strategy was to manipulate the structure of the lexicon by introducing a small set of words with illegal phonotactic patterning and examine how that affected phonotactic influences on speech perception in adult subjects with well-established phonological systems. Behavioral results showed that word learning significantly affected phonotactic repair. Neural analyses further suggested that the processing associated with these changes was consistent with the effects of word learning rather than rule learning. The underlying neurodynamic patterns were also consistent with those found in previous studies of phonotactic phenomena that did not depend on exposure to novel phonotactic structures (Gow and Nied, 2014; Gow and Olson, 2015). Together, these results support the hypothesis that top-down lexical influences on acoustic-phonetic processing drive perceptual phonotactic repair.

Our neural results do not implicate any dynamics or brain regions that are uniquely associated with rule learning or application, but it is possible that such processes co-localize with lexical or control processes. For this reason, it is important to consider the relationship between these phenomena and phenomena uniquely associated with the learning and application of rules. There is a large literature on phonotactic learning that shows that listeners can be induced to show sensitivity to artificial phonological distributional patterns after relatively short exposure to a set of nonword exemplars (see reviews by Moreton and Pater 2012a,b). Moreover, such exposure can influence both explicit metalinguistic judgments (Finley and Badecker, 2008) and implicit measures of performance, including naming accuracy and latency, recall, sensitivity and bias, and event-related potentials (Dell et al., 2000; Warker et al., 2008; Rossi et al., 2013; Bernard, 2015; Kittredge and Dell, 2016; Avcu and Hestvik, 2020). At the same time, evidence that even highly motivated adult bilinguals are unable to suppress first language phonotactic biases when speaking or perceiving a second language with different phonotactic constraints (Cutler et al., 1989; Freeman et al., 2016) suggests that there is a complicated relationship between these laboratory phenomena and natural phonological processes. Understanding this relationship is important, because the current results involve a laboratory manipulation affecting established natural language processing biases.

A meta-analysis by Anderson and Dell (2018) found that replicable effects of artificial first order phonotactic constraints (e.g., “/f/ must be a syllable onset)” on speech errors, with the learning of more complex constraints dependent on sleep consolidation. Unlike the current study, which introduced onset clusters that are disallowed or at least dispreferred in English (*/sr/ and */∫l/), the studies in Anderson and Dell’s meta-analysis all involve restrictions within a subset of allowed patterns (e.g., /f/ is allowed in both onset and coda position in English across vowel contexts). Studies of infants and children with weakly established phonotactic systems would seem to minimize conflict between existing and artificial systems. In this case, a meta-analysis by Cristia (2018) found that foundational findings by Chambers et al. (2003) involving rapid phonotactic learning do not replicate reliably across studies. Several studies have induced shifts in the processing of unattested or unlawful phonotactic structures, but all have involved either word learning (Ulbrich et al., 2016; Obrig et al., 2017; Wiese et al., 2017) or training involving the resyllabification of familiar English words (Whalen and Dell, 2006). We can find no clear independent evidence that artificial phonotactic training can induce shifts in the acceptability of unlawful forms in subjects’ native languages without word learning.

Evidence from studies that clearly isolate rule learning from word learning suggests that rule extraction depends on training features that were not present in the current experiment. Phonotactic learning paradigms typically depend on exposure to large sets of nonwords (see Moreton and Pater, 2012a,b for review). These paradigms may rely on explicit feedback during training (Pycha et al., 2003), explicit instruction to pay attention to the overall training set rather than individual words (Finley and Badecker, 2008), or training sets that lack unrelated filler items (Chambers et al., 2003). Rule extraction is also facilitated by modeling a novel pattern across a variety of speakers (Richtsmeier, 2011; Richtsmeier et al., 2011; Seidl et al., 2014), and many lexical contexts (Pierrehumbert, 2001; Hayes and Wilson, 2008; Richtsmeier, 2011; Denby et al., 2018). In contrast, the current experiment provided no feedback, specifically instructed subjects to learn words without making an explicit connection between word learning and later testing and modeled novel clusters in only 3/21 training words (<15%) using a single speaker.

On the contrary, our neural analyses suggest that the processes supporting phonotactic repair, and changes in the strength of phonotactic repair effects, are consistent with those found in previous studies of phonotactic repair in subjects who had not undergone language training of any kind. A similar set of language-related regions identified by data-driven algorithms in the current analysis (STG, SMG, angular gyrus, MTG, post central gyrus, and inferior temporal gyrus) was also identified by the same algorithm in study of phonotactic repair of Gow and Nied (2014) and study of phonotactic frequency effects of Gow and Olson (2015) in lexical decision. The effective connectivity analyses also replicated the primary findings of these earlier studies, which found a relationship between the strength of posterior middle temporal gyrus (pMTG) influences on the posterior STG (pSTG) and phonotactic effects. The current results also showed this effect and support the hypothesis that phonotactic influences on speech perception are lexically mediated.

Argument of Gow and Nied (2014) for the lexical mediation of phonotactic effects additionally rested on the finding that SMG influences on pSTG were stronger in trials in which subjects show behavioral evidence of phonotactic influences on speech categorization. The dual lexicon model (Gow, 2012) posits two phonological wordform areas: a ventral lexicon in pMTG that mediates the mapping between acoustic-phonetic and semantic or syntactic representation, and a dorsal lexicon in SMG that mediates the mapping between acoustic-phonetic and articulatory representations. We believe that the lack of a direct parallel effect of SMG on pSTG activation in the current study is a function of our word learning paradigm. The increased influence of pSTG on SMG in the Trained condition shows that word learning influenced SMG activation. While there was no increase in direct SMG influence on pSTG as a function of word learning, SMG clearly had indirect influences on pSTG through its influence on pMTG and the majority of ROIs that directly influenced pSTG.

More direct influences by SMG on pSTG were observed in post-hoc analyses comparing trials consistent with repair vs. in non-repair in the Naïve condition alone that replicated (Gow and Nied, 2014; see Supplementary Figure S1).

The consistency between the neural results of the current study and the previous studies of phonotactic phenomena using the same effective connectivity processing stream suggests that word learning interacted with existing processing mechanisms but did not introduce novel processes. The question then is whether learning words with phonotactically disallowed onset consonant clusters influenced phonotactic repair through lexical means alone, or by some combination of lexical and rule-mediated processes.

It is clear that lexical processes play some role in these results. Word learning is a lexical manipulation, and it influenced both behavioral and neural measures. Our results are consistent with several studies showing that word learning manipulations influence phonotactic sensitivity (Ulbrich et al., 2016; Obrig et al., 2017), or those showing that phonotactic constraints on processing are strengthened as a function of vocabulary size (Storkel, 2001; Edwards et al., 2004; Graf Estes et al., 2011).

Within the lexical mediation account of phonotactic processing, our results are also consistent with work demonstrating that word learning, especially when coupled with sleep consolidation, can influence lexical processing dynamics. For example, Gaskell and Dumay (2003) demonstrated that systematic exposure to the nonce word *cathedruke* produced competition effects on the recognition of its nearest phonological neighbor with a shared onset, *cathedral*, that were still measurable 1 week after exposure. This result has been widely replicated using lexical decision and visual world paradigm techniques in adult and child subjects (Magnuson et al., 2003a,b; Dumay and Gaskell, 2007; Kapnoula et al., 2015; James et al., 2017).

In summary, our behavioral and neural results support the hypothesis that phonotactic repair processes can be lexically mediated. Their consistency with previous effective connectivity analyses of phonotactic phenomena (Gow and Nied, 2014; Gow and Olson, 2015) involving native language phonotactic phenomena further suggests that lexical mediation is a general property of phonotactic phenomena. While we cannot rule out the hypothesis that rule or constraint learning contributed to our results, the rule hypothesis is not clearly supported by these or prior results. Future work should focus on determining the limits of lexical mediation as a driving mechanism in phonotactic phenomena.

## Data Availability Statement

The raw data supporting the conclusions of this article will be made available by the authors, without undue reservation, to any qualified researcher.

## Ethics Statement

The studies involving human participants were reviewed and approved by Partners Healthcare Review Board. The participants provided their written informed consent to participate in this study.

## Author Contributions

DG: conceptualization, stimulus design, project oversite and preparation of the original draft. AS: stimulus preparation and implementation. SA: scanning design. AS, EA, and SA: software and data analysis. DG, AS, EA, and SA: subject testing and writing – review and editing. All authors contributed to the article and approved the submitted version.

### Conflict of Interest

The authors declare that the research was conducted in the absence of any commercial or financial relationships that could be construed as a potential conflict of interest.

## Footnotes

^1^Quizlet.com

^2^https://quod.lib.umich.edu/cgi/c/corpus/corpus
