# Supplementary material for: Behavioral and Neurodynamic Effects of Word Learning on Phonotactic Repair
Source: Front Psychol. 2021 Mar 10;12:590155. doi: 10.3389/fpsyg.2021.590155 (PMC7987836; doi:10.3389/fpsyg.2021.590155)

Supplementary Material:

Table S1: Object names and photographs used in the word learning portion of the protocol. The pictures were presented with both a spoken and written form of the object name. The experimental stimuli critical for the study are indicated in bold face; however, all stimuli were presented in identical format to the subjects. Subjects were assigned to either sr- or shl- familiarization group and trained accordingly with List 1 or List 2.

	List 1	List 2
Stim 01	*shimboutch*	*wudgejev*
Stim 02	*chasdaid*	*thugneth*
Stim 03	*fausfied*	*thuzguithe*
Stim 04	*sathgace*	*shegzeeth*
Stim 05	*moomheak*	*yeeshgish*
Stim 06	*nyrleem*	*nezgeg*
Stim 07	*puzmape*	*thethyeesh*
Stim 08	*futneek*	*geethsedge*
Stim 09	*mishpook*	*lethfythe*
Stim 10	*teepshace*	*negsheg*
Stim 11	*shooltaid*	*chegyev*
Stim 12	*noomchale*	*theeshgythe*
Stim 13	*mowlral*	*thuvyep*
Stim 14	*chaiklud*	*theethchuz*
Stim 15	*gaidveet*	*chethzeeth*
Stim 16	*gubah*	*kusah*
Stim 17	*guboaf*	*kusoaf*
Stim 18	*gubeemp*	*kuseemp*
Stim 19	***sradex***	***shladex***
Stim 20	***sraspar***	***shlaspar***
Stim 21	***srigin***	***shlingin***

Figures

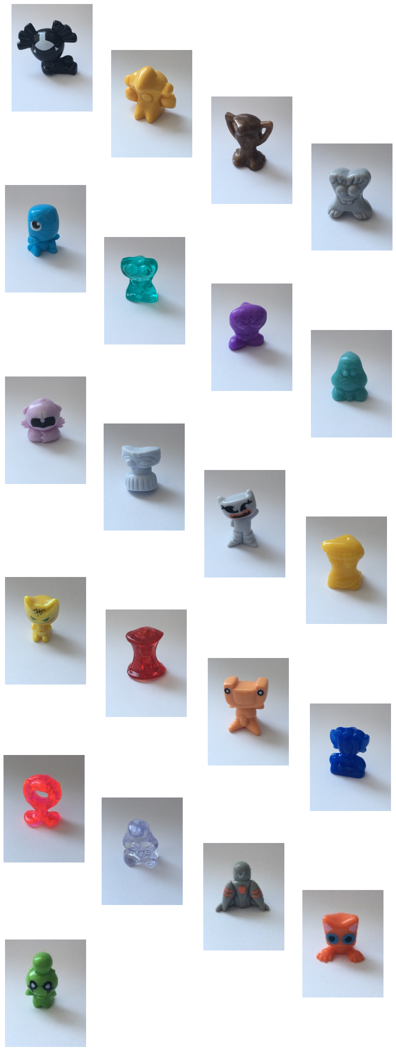

Table S2: **Regions of interest (ROIs) used in the Granger causation analyses.** MNI coordinates are given for the vertices showing the highest average activation across subjects within each ROI.

Label	Location	MNI Coordinates (X, Y, Z)		
**Left**				
AG1	Angular gyrus	-39	-67	45
ITG1	Inferior temporal gyrus (middle)	-56	-38	-21
ITG2	Inferior temporal area (anterior)	-48	-9	-36
Isth1	Isthmus of cingulate gyrus	-6	-38	6
LOC1	Lateral occipital cortex (inferior)	-15	-98	-16
LOC2	Lateral occipital cortex (anterior)	-45	-78	9
LOC3	Lateral occipital cortex (posterior)	-8	-102	8
MTG1	Middle temporal gyrus (anterior)	-63	-11	-17
MTG2	Middle temporal gyrus (posterior)	-64	-50	1
Medial1	Medial	1	0	12
ParsTri1	Pars triangularis	-48	35	-8
SFG1	Superior frontal gyrus (middle)	-11	63	16
SFG2	Superior frontal gyrus (inferior)	-6	42	46
SMG1	Supramarginal gyrus	-59	-50	23
SPC1	Superior parietal cortex (dorsal)	-11	-85	34
SPC2	Superior parietal cortex (ventral)	-14	-60	57
STG1	Superior temporal gyrus (posterior)	-68	-23	4
cMFG1	Middle frontal gyrus (caudal)	-42	15	47
postCG1	Postcentral gyrus (dorsal)	-14	-37	75
postCG2	Postcentral gyrus (middle)	-52	-21	52
postCG3	Postcentral gyrus (ventral)	-62	-10	29
preCG1	Precentral gyrus (ventral)	-57	5	13
**Right**				
AG1	Angular gyrus	42	-57	44
ITG1	Inferior temporal gyrus (anterior)	48	-11	-38
ITG2	Inferior temporal cortex (posterior)	55	-55	-18
LOC1	Lateral occipital cortex (posterior)	23	-99	-8
LOC2	Lateral occipital cortex (anterior)	46	-80	-3
MTG1	Middle temporal gyrus (middle)	65	-33	-9
ParsTri1	Pars triangularus	52	29	2
SFG1	Superior frontal gyrus (posterior)	12	-3	69
SFG2	Superior frontal gyrus (posterior)	18	13	61
SPC1	Superior parietal cortex (inferior)	21	-82	39
SPC2	Superior parietal cortex (superior)	15	-64	61
STG1	Superior temporal gyrus (posterior)	63	-38	13
TPol1	Temporal pole	31	18	-33
postCG1	Postcentral gyrus (middle)	45	-21	57
postCG2	Postcentral gyrus (ventral)	63	-10	14
postCG3	Postcentral gyrus (superior)	12	-33	76
rMFG1	Middle frontal gyrus (rostral)	20	61	0

Figure S1: **Analysis of differential influences on left posterior superior temporal gurys (pSTG) by the other ROIs in trials producing phonotactic bias-consistent (legal, "Repaired") vs. bias-inconsistent (illegal, "Unrepaired") phoneme categorization.** The target ROI (left pSTG) is shown in red. Bubble size indicates the relative strength of Granger influences (number of time points that show GCI values > 0.20 within 200-400 ms post-stimulus-onset time window. Regions with stronger (weaker) influences in bias-consistent than bias-inconsistent trials are shown in green (orange). The analysis was limited to trials in the Naïve condition only, which reduced the power of the analysis; nevertheless, this result replicates the association between phonotactic repair and significant top-down influences by left supramarginal gurys (SMG) on left pSTG found by Gow and Nied (2014) using the same paradigm and stimuli. In both studies $p < 0.05$ after FDR correction for multiple comparisons (Benjamini & Hochberg, 1995).

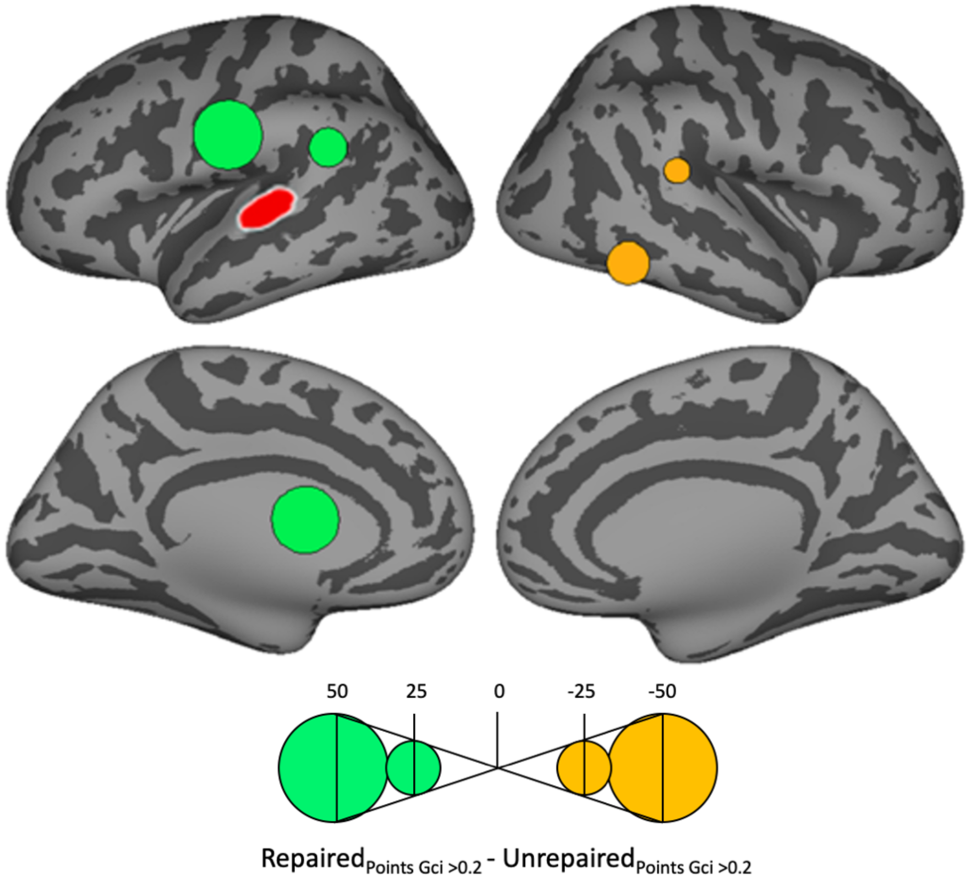

Supplement: Supplementary file 1 [file Data_Sheet_1.pdf]
